# Chemical Pollutants of Pharmaceutical Origin Present in the Environment

**DOI:** 10.5696/2156-9614-8.19.180916

**Published:** 2018-09-10

**Authors:** Lilian Corra

**Affiliations:** Director, Master of Information on Health and Environment, School of Medicine, University of Buenos Aires. Argentina.; Director, Medical Specialist Program on Health and Environment, School of Medicine, University of Buenos Aires. Argentina; Government Relations Advisor, South America, Pure Earth; President, International Society of Doctors for the Environment - ISDE

## Abstract

Competing Interests. The author declares no competing financial interests.

**Figure:**
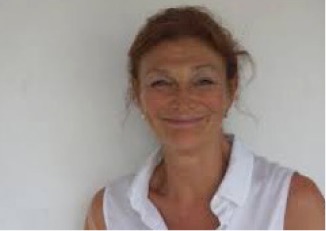


Chemicals of pharmaceutical origin, independent of their origin, behave as pollutants when they enter the environment. They may have negative effects and impacts on the environment, affecting biodiversity as well as human health.

Detection of these chemicals when present in surface water is not a recent finding, but the growing number of publications on their detection in the environment and the scientific evidence of their possible negative effects on microorganisms, wildlife and human health raise important questions.

The main questions are:

- How much do we know about the presence of chemicals of pharmaceutical origin in the environment?
- Which of these chemicals or their sub-products are persistent and how long do they persist?
- Are they also present in drinking water? To what extent?
- What are the effects on microorganisms, wildlife and human health of chronic exposure to low doses of these chemicals?
- How they behave in the environment?
- Do they bio-accumulate, bio-concentrate and bio-magnify?
- How toxic are early exposures following conception?
- How toxic (or cytotoxic) are pharmaceuticals created/designed to act as hormones, anesthetics, mayor analgesics, and psychoactive drugs?
- How do they affect the environmental microbiology balance (microorganisms)?
- What are the effects on flora or microflora?
- And so on… there are still many questions and gaps to be explored.

To advance discussion on this topic, lines of research and information exchange have been organized to explore and prioritize an innovative approach to analyze this problem. This will allow us to better examine chemicals of pharmaceutical origin present in the environment as pollutants, independent of their origin, integrating previous experiences with pollutants from industrial origins.

Chemicals of pharmaceutical origin present in the environment may have the same toxic characteristics of chemicals from industrial sources. Some pharmaceutical chemicals are designed to have specific and defined effects. However, other pharmaceutical drugs (synthetic or non-synthetic) have mechanisms of action which are not well known.^1^

The health effects of environmental pollution are not often examined in a holistic manner. The behavior and health effects of pharmaceutical chemicals as environmental pollutants are not well known and may differ from the health effects of individual chemicals in their role as pharmaceuticals and from their effects on other living things (microorganisms and wildlife). Furthermore, little is known of their effects on plants, microflora and flora in general.

In general, the effects of pharmaceuticals when present since conception are rarely studied, and research on early exposures is scare.

Attention has been focused on antimicrobial resistance created by the presence of antibiotics in the environment. As this emerging problem poses a new and unexpected challenge to human and environmental health, this approach may lead to distortion and partial evaluation of the problem, delaying the implementation of effective and timely interventions.

## Where to begin with regulation and control of pollution from chemicals of pharmaceutical origin?

To be consistent with the analysis of other chemical pollutants present in the environment, the concept of cradle to cradle, circular economy or green economy should be applied.

Analyses are needed focusing on the production of supplies, manufacturing, marketing (organization of demand, purchasing), packaging and final disposal (both of industrial waste as well as urban and/or hospital waste streams that contain these chemicals, both in solid and liquid form). In addition, examination is needed of the various methodologies used to treat pharmaceutical-containing waste at the end of their useful life.

To facilitate this process, it is necessary to have access to information. The pharmaceutical industry, however, guards this information closely as their main objective is health care and the main financial interest involves developing scientific and technological innovation (knowledge), resulting in a strong policy of protection of information.

As needed information may not be completely available, the implementation of effective emissions control measures of related industrial processes are difficult to evaluate. Similarly, limited access to information on the nature and mechanisms of pharmaceutical chemicals hinders a full understanding of their behavior in the environment and their possible effects on human health and the environment. This lack of information can also influence analyses and selection of the Best Available Techniques and Best Environmental practices (BAT/BEP) for the final disposal of waste all along their life cycle.

We are clearly facing an issue that forces us to think differently, to broaden our knowledge and position on the nature of chemicals that are emitted to the environment and their possible effects on the environment, living organisms and human health.

Efforts are needed to raise the visibility of this new and emerging issue and to begin discussion and analysis of the current situation. Focus should be placed on determining its scope, and in particular, on how to develop and implement effective interventions and measures to prevent pollution, in order to reduce the dumping and emissions of chemicals of pharmaceutical origin into the environment and their negative effects.

## Contaminantes Químicos de Origen Farmacéutico Presentes en el Ambiente

Los químicos de origen farmacéutico, independientemente de su origen, se comportan como contaminantes cuando entran al ambiente. Pueden tener efecto negativo sobre el ambiente, la biodiversidad y la salud humana.

La presencia de estos químicos en el agua superficial no es un hallazgo reciente ya que existe un número creciente de publicaciones sobre su detección y evidencia científica de los posibles efectos negativos sobre los microorganismos, la vida silvestre y la salud humana lo que plantea preguntas importantes. Las principales podrían ser:

- ¿Cuánto sabemos sobre la presencia de los químicos de origen farmacéutico en el ambiente?
- ¿Cómo se comportan en el ambiente?
- ¿son persistentes?, ¿cuánto tiempo persisten?
- ¿Se bio-acumulan, bio-concentran y bio-magnifican?
- ¿En que medida están presentes en el agua de bebida?
- ¿Cuáles son los efectos de la exposición crónica a bajas dosis sobre microorganismos, vida silvestre y salud humana?
- ¿Qué tan tóxica es la exposición temprana desde la concepción?
- ¿Qué tan tóxicos son los diseñados para actuar como hormonas, anestésicos, analgésicos, psicodrogas o citotóxicos?
- ¿Cómo afectan el equilibrio microbiológico ambiental?
- ¿Cuáles son los efectos sobre la flora o la microflora?

Y así sucesivamente ... todavía quedan muchas preguntas que responder y lagunas de información por explorar.

Para avanzar en la discusión sobre este tema, se han organizado líneas de investigación e intercambio de información existente sobre la naturaleza del problema para explorarlo, priorizarlo e integrarlo desde un punto de vista innovador tomando en consideración su génesis.

El origen (farmacéutico) de estas sustancias propone una arista nueva al problema de contaminación ambiental química. Se puede percibir que la naturaleza de su origen influye en la comprensión y el abordaje holístico del problema, funciona como una barrera para parangonarlos con los contaminantes químicos de origen industrial. Evidentemente y de igual manera son contaminantes indeseados del ambiente, independientemente de su origen y como tales deben ser estudiados y tratados.

Los químicos de origen farmacéutico presentes en el ambiente podrían tener las mismas características tóxicas que los procedentes de fuentes industriales. Algunos están diseñados para tener efectos específicos, definidos y bien conocidos (hormonas y antibióticos, por ejemplo). Sin embargo, otros fármacos (sintéticos o no sintéticos) tienen efectos derivados de mecanismos de acción que no son bien conocidos.^2^

Los efectos de la contaminación ambiental no suelen examinarse de manera holística. El comportamiento en el ambiente y sus efectos actuando como contaminantes no son bien conocidos. Los efectos sobre la salud humana de la exposición a químicos de origen farmacéutico (exposición que puede ser múltiple y simultanea) puede diferir de los buscados cuando son administrados individualmente en un tratamiento médico, o de los efectos sobre otros seres vivos (microorganismos y vida silvestre). Además, poco se sabe de sus efectos sobre las plantas, la micro-flora y la flora en general.

En general, rara vez se estudian o tienen conocimientos sobre los efectos de los químicos de origen farmacéuticos cuando están presentes desde el momento mismo de la concepción, la investigación sobre la exposición temprana in utero en general es muy limitada.

Se ha centrado mucho la atención en la resistencia creada por los antimicrobianos presentes en el ambiente. Este importante problema emergente plantea un nuevo e inesperado desafío para la salud humana y ambiental, pero debemos ser conscientes de que destacar solo este perfil puede conducir a una distorsión y la evaluación parcial del problema, retrasando la implementación de intervenciones efectivas, oportunas y certeras en el tema.

### ¿Por dónde comenzar con la regulación y el control de la contaminación por químicos de origen farmacéutico?

Para ser coherente con el análisis de otros contaminantes químicos presentes en el ambiente, se debe aplicar el concepto de la cuna a la cuna, la economía circular o economía verde.

Se necesitan análisis centrados en la producción de suministros, fabricación, comercialización (organización de la demanda, compras), envasado y disposición final (tanto de residuos industriales como de residuos urbanos y / o hospitalarios que contienen este tipo de químicos, tanto sólidos como líquidos). Además, es necesario examinar las diversas metodologías utilizadas para tratar los desechos que contienen químicos farmacéuticos al final de su vida útil.

Para facilitar este proceso, es necesario tener acceso a la información. La industria farmacéutica, sin embargo, guarda esta información muy celosamente ya que su principal objetivo es la atención médica y su interés financiero implica el desarrollo competitivo de innovación científica y tecnológica (conocimiento), lo que resulta en una política sólida de protección de la información que se aplica en todo el mundo.

Como la información puede no estar completamente disponible, la implementación de medidas efectivas de control de emisiones derivadas de los procesos industriales relacionados a la actividad es difícil de evaluar. Del mismo modo, el acceso limitado a la información sobre la naturaleza y los mecanismos de los químicos farmacéuticos dificulta una comprensión integral de su comportamiento en el ambiente y sus posibles efectos sobre la salud humana y las demás especies. Esta falta de información también puede influir en los análisis y la selección de las Mejores Técnicas Disponibles y las Mejores Prácticas Ambientales (BAT / BEP) para la eliminación final adecuada de los desechos a lo largo de su ciclo de vida.

Claramente nos enfrentamos a un problema que nos obliga a pensar de forma diferente, a ampliar nuestro conocimiento y nuestra posición sobre la naturaleza de los químicos que se emiten al ambiente y sus posibles efectos sobre el equilibrio del mismo ambiente, la salud humana y de los demás organismos vivos.

Se necesitan centrar los esfuerzos en aumentar la visibilidad de este tema nuevo y emergente promoviendo la discusión y el análisis de la situación actual. Urge determinar el alcance y caracterizar el problema para desarrollar e implementar intervenciones efectivas de prevención de la contaminación reduciendo el vertido y emisiones de químicos de origen farmacéutico al ambiente y sus efectos negativos.
